# *Haemophilus parainfluenzae* Mural Endocarditis: Case Report and Review of the Literature

**DOI:** 10.1155/2016/3639517

**Published:** 2016-06-12

**Authors:** Luca T. Giurgea, Tim Lahey

**Affiliations:** ^1^Department of Medicine, Dartmouth-Hitchcock Medical Center, Lebanon, NH 03756, USA; ^2^Section of Infectious Disease and International Health, Department of Medicine, Dartmouth-Hitchcock Medical Center, Lebanon, NH 03756, USA

## Abstract

*Haemophilus parainfluenzae*, which uncommonly causes endocarditis, has never been documented to cause mural involvement. A 62-year-old immunocompetent female without predisposing risk factors for endocarditis except for poor dentition presented with fever, emesis, and dysmetria. Echocardiography found a mass attached to the left ventricular wall with finger-like projections. Computed tomography showed evidence of embolic phenomena to the brain, kidneys, spleen, and colon. Cardiac MRI revealed involvement of the chordae tendineae of the anterior papillary muscles. Blood cultures grew* Haemophilus parainfluenzae*. The patient was treated successfully with ceftriaxone with resolution of symptoms, including neurologic deficits. After eleven days of antibiotics a worsening holosystolic murmur was discovered. Worsening mitral regurgitation on echocardiography was only found three weeks later. Nine weeks after presentation, intraoperative evaluation revealed chord rupture but no residual vegetation and mitral repair was performed. Four weeks after surgery, the patient was back to her baseline. This case illustrates the ability of* Haemophilus parainfluenzae* to form large mural vegetations with high propensity of embolization in otherwise normal cardiac tissue among patients with dental risk factors. It also underscores the importance of physical examination in establishing a diagnosis of endocarditis and monitoring for progression of disease.

## 1. Introduction

Endocarditis is a rare but serious infection with hospital mortality averaging 18% but dependent upon causative pathogen, lesion type, and patient comorbidities [1]. Mural endocarditis, seen in 4% of cases of endocarditis, is defined as inflammation of the nonvalvular endocardial surface in any of the four chambers of the heart [2]. It is thought to arise from seeding of either congenitally or iatrogenically abnormal endocardium.* Staphylococcus aureus* and streptococci are the most common causes of mural endocarditis, whereas mural endocarditis from the HACEK organisms (*Haemophilus* species,* Aggregatibacter *species,* Cardiobacterium hominis*,* Eikenella corrodens, *and* Kingella *species) has not been reported [2, 3], even though 1.4% of total endocarditis cases are attributed to HACEK organisms [4].

## 2. Case Description

A 62-year-old female with a history of migraines and hypertension presented with nausea, malaise, and vertigo. Twelve days prior to admission, she developed discomfort and pressure in the epigastric region. Five days later, she developed night sweats, chills, and emesis and presented to an outside hospital. She was given intravenous fluids with potassium and sent home. Her symptoms persisted and, two days prior to admission, she developed vertigo and light headedness. She presented back to the outside hospital. Vital signs were within normal limits. Laboratory data revealed a leukocytosis of 13.3 × 10^3^/*μ*L and mildly elevated troponin. Computed tomography (CT) of the head showed a 2 cm right occipital region of diminished attenuation of uncertain acuity. Blood cultures were drawn and she was transferred to our hospital. On presentation, she also complained of bifrontal headache different from her usual migraine, flashes of light, and subtle left peripheral vision loss. She had no history of congenital cardiac disease, rheumatic fever, or IV drug use. Neurological examination was remarkable for somnolence, generalized weakness, mildly decreased left peripheral vision, and dysmetria. Cardiovascular exam revealed no murmur or gallop. Abdominal exam was unremarkable. Poor dentition was noted. Blood and urine cultures were drawn and ceftriaxone and vancomycin were started. On hospital day (HD) 2, dysmetria worsened and she spiked a fever of 39°C. Splinter hemorrhages were noted. Transthoracic echocardiography (TTE) showed a mobile mass in the left ventricle. Magnetic resonance imaging (MRI) of the head showed cerebellar, parietal, and occipital lesions. CT of the abdomen and pelvis revealed colonic inflammation, hepatomegaly, and multiple renal and splenic infarcts. HIV testing was negative. After 23 hours, blood cultures from this hospital grew small gram negative coccobacilli. Vancomycin was discontinued. By HD 3, cultures from the outside hospital grew gram negative coccobacilli as well. Transesophageal echocardiography (TEE) showed a complex echogenic mass of 1.5 cm by 1.7 cm in the left ventricle, which extended into the left ventricular outflow tract with finger-like projections. A small filamentous vegetation was noted on the anterior leaflet of the mitral valve. By HD 4, dysmetria and malaise improved. The isolate growing in blood cultures was identified by biochemical testing as* Haemophilus parainfluenza*e susceptible to ceftriaxone. Subsequent cultures on antibiotics remained negative. Cardiac MRI showed two mobile nodular structures located near the chordae tendineae of the anterior papillary muscle along its posterior aspect and near the anterior mitral valve leaflet. Repeat MRI of the brain with diffusion weighted imaging showed new infarcts within the cerebellum and cerebral hemispheres. On HD 11 she developed a faint systolic murmur, which worsened rapidly. By HD 12, it became a holosystolic murmur loudest at the apex, radiating to the axilla. Surgery was considered but repeat TEE demonstrated a substantial decrease in vegetation size and only mild mitral regurgitation which was unchanged from the prior study. The patient was discharged to complete four weeks of ceftriaxone. At follow-up two months later, she was doing well without any neurological deficits and no signs or symptoms of heart failure. Repeat TEE showed resolution of vegetations but residual severe mitral regurgitation. She underwent mitral valve repair without further complications. At follow-up 4 weeks after surgery, she was back to her baseline health, without any dyspnea on exertion or neurologic sequelae.

## 3. Discussion

*Haemophilus parainfluenzae* is a rare cause of subacute endocarditis, particularly in North America [1, 5].* H. parainfluenzae* is responsible for 0.5% of total endocarditis cases and 36% of HACEK endocarditis cases [4]. Poor dentition is seen in as many as 20% of patients afflicted with HACEK endocarditis, with nearly twice that many patients also having recently undergone a dental procedure [6, 7]. The vegetations in* Haemophilus *spp. endocarditis are classically large and have a high risk of embolization, at 35.7% among all* Haemophilus* spp. but up to 60% in cases of endocarditis specifically attributed to* Haemophilus parainfluenzae *[6, 7]. The risk of embolization is associated with large vegetation size and hyphal morphology of vegetations in* Haemophilus* endocarditis [8]. In a study of all cause endocarditis, vegetations greater than 10 mm exhibited 2.8 greater odds of embolization [9]. More than half of patients with* Haemophilus parainfluenzae* endocarditis have no predisposing valvular disease, although congenital and iatrogenic abnormalities of the myocardium are common as among all cases of endocarditis [8]. The mitral valve is most commonly affected in* Haemophilus *endocarditis followed by the aortic valve [7].* Haemophilus* can be difficult to grow in vitro and susceptibilities may be unavailable. Due to significant presence of ampicillin resistance, ceftriaxone is often used unless susceptibilities dictate otherwise. Therapy usually involves 4 weeks of intravenous antibiotics in native valve endocarditis and 6 weeks in prosthetic valve endocarditis [10].

A rare presentation of endocarditis, mural endocarditis can involve the endocardium in both atria and ventricles [11] and most commonly involves previously abnormal endocardium affected by mural thrombi, myocardial abscesses, pacemaker lead sites, congenital defects, hypertrophic subaortic stenosis, jet lesions, ventricular aneurysms, or pseudoaneurysm [3]. Immunocompromise from chemotherapy, steroids, and other immunosuppressant medications underlies many cases of mural endocarditis, particularly those caused by fungal organisms [3]. Organisms commonly implicated in mural endocarditis include staphylococci, streptococci,* Enterococcus* spp.,* Salmonella* spp.,* Klebsiella* spp.,* Bacteroides fragilis*,* Candida* spp., and* Aspergillus* spp. [3]. Despite cases series [4, 6–8] on HACEK endocarditis including one published by Chambers et al. in 2013 on 77 patients and one published by Das et al. in 1997 on 45 patients, we are aware of no reports of mural endocarditis attributed to HACEK organisms.

The patient in this case had a very small mitral vegetation and initial echocardiography only showed trace mitral regurgitation, so it was unsurprising that there were no auscultatory findings early on [12]. Although not specifically addressed in larger studies, other case reports of mural endocarditis have also reported absence of murmur [13, 14]. Our patient developed severe mitral regurgitation after discharge and was found to have chordae tendineae rupture along with an audible murmur. Cardiac MRI did show involvement of the chordae tendineae by the mural vegetation. It is interesting to note that despite echocardiography's documented superior sensitivity in diagnosing mitral regurgitation in comparison with physical examination [12], the development and worsening of a murmur on cardiac auscultation in this patient heralded deterioration of mitral valve function not immediately identifiable on echocardiography.

## 4. Conclusion

*Haemophilus parainfluenzae* is an uncommon cause of endocarditis that affects patients with poor dentition and causes large vegetations with a high propensity for embolization. It can affect both normal and abnormal valves and can rarely cause mural vegetations which may be silent on cardiac examination. Careful auscultation for changes in murmurs can reveal deterioration of affected valve function, which may appear unchanged on echocardiography. Antibiotic therapy alone can be effective but early surgical intervention should be considered for patients with large vegetations or persistent embolization despite appropriate therapy.
